# Bibliographical

**Published:** 1886-03

**Authors:** 


### BIBLIOGRAPHICAL.

NOTES ON ANÆSTHETICS, *with an appendix containing illustrative cases and engravings of Anæsthetic apparatus.* By ARTHUR S. UNDERWOOD, M. R. C. S., L. D. S., Eng. Lecturer on Dental Anatomy and Physiology, and Assistant Surgeon at the Dental Hospital, London, etc. London: Claudius Ash & Sons. 1885.

The editor of *The Journal of the British Dental Association* is not unknown upon this side of the Atlantic ocean. He is a terse and vigorous writer, and the volume here presented is not unworthy of him. In the preface he modestly disclaims consideration as an authority, and announces that to Mr. Woodhouse Brain has been submitted the manuscript, for any needed corrections.

The directness with which he approaches his subject, the absence of circumlocution and rhetorical display are characteristic of the writer, and much to be commended. When, in a work upon any scientific or professional subject, the author introduces irrelevant matter and indulges in discussive disquisitions, the argument is clogged and the point is apt to be entirely lost. A style too much condensed is, on the other hand, apt to degenerate into dogmatism and arrogance of assertion. From both these faults the book under notice is remarkably free. In the main, the teachings will commend themselves to any intelligent man, although there are some assertions which we cannot permit to pass without question.

In speaking of Nitrous Oxide Gas, the author says :

"The risk to life is so small, that it may safely be said that, supposing a cardiac condition existed that rendered Nitrous Oxide Gas a dangerous agent, in such a case any operation, even the extraction of a tooth, *without an anæsthetic*, would be attended with still greater danger. To put the case in other words, any short operation becomes less dangerous to life when performed under gas than when the anæsthetic is not employed."

We do not think that cardiac complications so urgently contra-indicate the use of Nitrous Oxide as certain conditions of blood-tension, or cerebral disturbances. The heart is not so unduly stimulated as in the administration of chloroform, but if the anæsthesia be very profound, the vaso-motor system may be seriously disturbed. Nor do we think it well to resort to the use of an anæsthetic for every trivial operation. Even the temporary suspension of the function of a great part of the nervous system is a matter of grave import, and not to be entered upon without due and adequate cause.

The author's remarks upon the administration of chloroform are very just, and the great source of danger, the administration of an atmosphere too highly charged with the vapor, is clearly pointed out.

The chapter upon the Physiology of Anæsthesia does not comport with the simple character of the remainder of the book. Fourteen pages of a work like this are scarcely sufficient to make plain the full physiological action of so powerful a class of drugs as the anæsthetics, and less than this is provokingly unsatisfactory. But its reading will stimulate a desire for further information, and thus lead to great good.

As a hand-book for students and others, Mr. Underwood's book will occupy an honorable place in our literature, and it would be better for dentistry if it could be placed in the hands of every practitioner.

LECTURES ON SYPHILIS. *Delivered at the Chicago College of Physicians and Surgeons.* By G. FRANK LYDSTON, M. D., late resident surgeon at Charity hospital, and at State Emigration Refuge and Hospital, New York City. Lecturer on Surgical Diseases of the Genito-Urinary Organs and on Venereal Diseases in the College of Physicians and Surgeons, etc., etc. Chicago: A. M. Wood & Co. 1885.

This series of lectures was originally published in the *Western Medical Reporter*, where they met with such favor as to call for the republication in a separate form. They give a plain and practical idea of the nature and character of this terrible disease, and as such commend themselves to all who follow the healing art.

The book will be especially useful to dentists, for it is urgently necessary that they should be able to recognize the characteristic lesions produced by syphilis in the oral cavity. There is a possible danger of carrying infection upon instruments used for syphilitic patients, unless extreme caution be exercised, and the dentist should therefore be able, at least, readily to recognize such cases, that he may take the necessary precaution. A dental education is not supposed to include the pathology of all the general disturbances of the body, and it becomes the duty of every earnest dentist to supplement his college course by the careful study of works upon general medicine. Few subjects will prove more interesting or useful to him than a careful consideration of venereal disorders, as their complications may seriously involve his specialty. We can heartily commend this book to all in search of such information.

DIAGRAM OF PARLIAMENTARY RULES, *with a key, together with concise hints and directions for conducting the business of deliberative assemblies.* By Uriah Smith, Battle Creek, Mich. Review and Herald Publishing Association. 1885.

Every attendant upon dental associations is desirous of knowing the parliamentary rules which should govern the deliberations. The manuals of Jefferson and of Cushing have been accepted as authorities in the decision of questions of order. But unless there has been exhaustive study of their contents, sufficient to make familiar to the mind every established rule, much time must be spent in looking up the authority. Without unduly delaying proceedings and interrupting the progress of business, this is impossible. The admirable diagram of Mr. Smith presents to the eye at once all the rules which in parliamentary practice apply to any motion, and enables a presiding officer to decide instantly any question that may arise, with the certainty that he is correct.

CAULK'S DENTAL ANNUAL. *Number IV.* 1885-1886.

This admirable collection of dental statistics arrived too late for notice in the January number, but it is not now too late to commend it to every dentist. It contains a directory of dental societies, of dental periodicals, of dental colleges, number of dentists, of dealers in dental goods, of dental patents, transcripts of the dental laws of the different States, dental necrology, with original articles and notes that make it almost a necessity to every intelligent dentist. L. D. Caulk, Camden, Del., Publisher. Price 25 cents.

ERYTHROXYLON COCA, *and its derivatives.* A *resumé* of their history, botanical origin, production and cultivation, chemical composition, therapeutic application, physiological action and medicinal preparation. Compiled by the scientific department of Parke, Davis & Co. Detroit and New York.

Muriate of Cocaine has sprung into such universal use in medicine that some knowledge of its character is desired by every physician. In its preparation no one has reached a greater degree of perfection than the well known manufacturing chemists, Parke, Davis & Co. We have for some time been using their preparations of Cocaine, the muriate, the oleate, the salicylate, the hydrobromate, and the citrate, and always with increasing satisfaction. This pamphlet of one hundred pages is devoted to a thorough consideration of the properties and effects of cocaine in the treatment of a great variety of disorders. Its adaptation to dental practice is clearly set forth, and we heartily recommend its study to every progressive dentist. The pamphlet may be obtained of the publishers.

PRACTICAL NOTES ON THE TREATMENT OF SKIN DISEASES. *Part I. Diseases of the Perspiratory and Sebaceous Glands.* By George H. Rohe, M. D., Professor of Hygiene and Clinical Dermatology in the College of Physicians and Surgeons of Baltimore. Baltimore: 1885.

The well known author of " A Text Book of Hygiene," and former editor of *The Medical Chronicle,* needs no introduction to our readers. He is always concise, instructive and reliable. This little book of sixty pages contains all that it is essential to know of the subjects of which it treats. It may be obtained of the author at Baltimore. Price 25 cents.

TRANSACTIONS OF THE DENTAL SOCIETY OF THE STATE OF NEW YORK. *Seventeenth Annual Meeting*, 1885.

The annual volume of this society is somewhat late in making its appearance, and is less full and complete than most of its predecessors. But it perhaps contains all that it was worth while to insert. Some of the papers are of permanent value. The work is well done and the report makes a handsome volume.

TRANSACTIONS OF THE MEDICAL SOCIETY OF THE STATE OF PENNSYLVANIA. *Thirty sixth Annual Session, at Scranton, May,* 1885.

*Report of the Board of Dental Examiners of the State of California.*

*First Annual Report of the Minnesota State Board of Dental Examiners.*

*Diphtheria and its Management.* Are Membraneous Croup and Diphtheria Distinct Diseases? BY JOSEPH E. WINTERS, M. D. Reprinted from *The Medical Record.*

*Joint Diseases.* Treatment by Rest and Fixation. BY DE FOREST WILLARD, M. D. Reprinted from *The New York Medical Journal.*

*Surgical Treatment of Infants.* BY DE FOREST WILLARD, M. D.

*Color Blindness.* A paper read before the Buffalo Society of Natural Sciences. BY B. H. GROVE, A. B., M. D.

*Clinical Notes on the Local Treatment of Disease.* A record of practical therapeutics. Edited by Charles L. Mitchell, M. D.

*Editorial Reprints from the Journal of Inebriety.* By T. D. Crothers, editor.

*Angioma of the Nose.* BY JOHN O. ROE, M. D. Reprinted from *The New York Medical Journal.*

*Bronchietasis.* BY JOHN O. ROE, M. D. Reprinted from *The Buffalo Medical and Surgical Journal.*

*An Essay on Vulcanizing Rubber.* BY FREDERICK WHEATON SEABURY, Providence, R. I.

